# Sustainable funding for biocuration: The Arabidopsis Information Resource (TAIR) as a case study of a subscription-based funding model

**DOI:** 10.1093/database/baw018

**Published:** 2016-03-17

**Authors:** Leonore Reiser, Tanya Z. Berardini, Donghui Li, Robert Muller, Emily M. Strait, Qian Li, Yarik Mezheritsky, Andrey Vetushko, Eva Huala

**Affiliations:** Phoenix Bioinformatics, The Arabidopsis Information Resource, 643 Bair Island Rd. Suite 403, Redwood City, CA 94063, USA

## Abstract

Databases and data repositories provide essential functions for the research community by integrating, curating, archiving and otherwise packaging data to facilitate discovery and reuse. Despite their importance, funding for maintenance of these resources is increasingly hard to obtain. Fueled by a desire to find long term, sustainable solutions to database funding, staff from the Arabidopsis Information Resource (TAIR), founded the nonprofit organization, Phoenix Bioinformatics, using TAIR as a test case for user-based funding. Subscription-based funding has been proposed as an alternative to grant funding but its application has been very limited within the nonprofit sector. Our testing of this model indicates that it is a viable option, at least for some databases, and that it is possible to strike a balance that maximizes access while still incentivizing subscriptions. One year after transitioning to subscription support, TAIR is self-sustaining and Phoenix is poised to expand and support additional resources that wish to incorporate user-based funding strategies.

**Database URL**: www.arabidopsis.org

## Introduction

Biological science is increasingly a quantitative, data intensive, data driven enterprise. Databases and data repositories serve essential functions of making data widely available, searchable, retrievable, and persistent. Biocurators, who organize and annotate the data, ensure that information is well described, structured and discoverable. Although the number of databases is increasing, the funding available to support them has not kept pace, leading to a sustainability crisis (1–4). The primary source of funding for most databases comes from competitive grants for which established databases must continually compete alongside new projects. With grant cycles typically lasting 3–5 years, for any given database, the prospects for long-term funding are uncertain. In a recent assessment of long-term survivability of databases, 62.3% of 326 databases listed in the 1997 DBcat were considered ‘dead’ after 18 years (4). Survivors tended to have strong, long-term sources of financial support (i.e. institutional or direct government funding) whereas extinct, ‘zombie’ or dying databases tend to have weaker, transient financial supports such as grants. Because the primary emphasis in government research funding is on innovation and novel research, and budgets are limited (5), it is becoming increasingly challenging to find grants to support essential database services such as maintenance and ongoing data curation. Even when databases can obtain renewals, they may experience a decrease or transient gaps in funding that can lead to staff turnover. This, in turn can have a negative impact on those resources when experienced people leave. When funding is cut back or lost altogether, databases are forced to curtail activities such as curation, become static or shut down entirely. The effect on research productivity can be significant when databases disappear or can no longer integrate up to date, relevant information. Clearly, the existing funding paradigm for databases leaves much to be desired. One of the challenges created by ‘big data science’ is supporting the expanding digital data ecosystem, including finding long-term stable funding for community databases (5).

To address this sustainability crisis, several alternative funding models have been proposed (6–9). In the infrastructure model, governments or institutions set aside funds explicitly for long-term funding of digital data. This model has great potential to ensure both sustainability and data accessibility; however, this requires a considerable, long- term political and financial commitment on the part of government or institutional entities. Another proposed model is to allocate a pool of competitive grant funds specifically for continuing support of existing databases and repositories. To be effective, both of these funding models must be based on metrics that measure a resource’s utility and importance to researchers, rather than degree of innovation, which is frequently the main criterion for funding for existing grant-based models. If the goal is to support research, database innovation is only useful to the extent that it provides better service to the research community. Established software tools and methods frequently provide more robust and cost-effective solutions for infrastructure needs than more innovative but unproven new technologies, and infrastructure projects should be encouraged to choose a mix of new and proven methods that provide the best service for the lowest cost.

**Figure 1.**
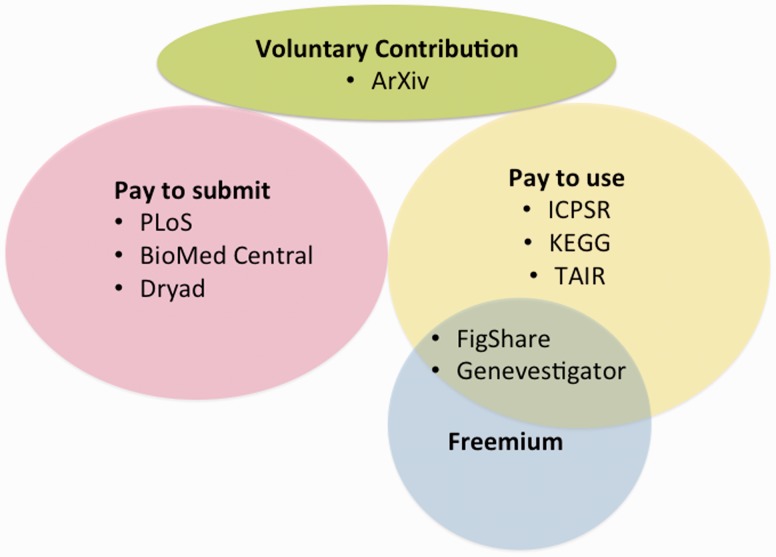
User fee-based funding models. The three main types of user fee models and variations. With the pay to submit model, data are open access and users pay to publish or deposit data. In the pay per use model, uses must pay a fee for data access. User fees can take the form of memberships (ICPSR) or subscriptions. The ‘freemium’ is a hybrid pay for use model in which data access is free but users pay a premium for additional services. Voluntary contributions allow for the broadest data access.

The two models described earlier share the disadvantage that the funds may be drawn from the already limited pool of funding available for research. An alternative that avoids tapping limited government research budgets, and offers an inherent metric of usefulness to researchers, is to distribute the costs to users, either by charging for data submission or data access or requesting voluntary contributions (Figure 1). These different user fee models each have advantages and disadvantages. The fee for data submission model, exemplified by the open access publisher Public Library of Science (PLoS), has been widely adopted by open access journals. Access to articles is made free to users and supported by fees charged to authors. This is the model adopted by the Dryad data repository (http://datadryad.org/). Dryad stores data sets from publications and charges a fee either to the authors or publishers of the original publication. The fees cover basic metadata curation, validation and assignment of DOIs (digital object identifiers) to datasets. For a pay to deposit model to be sustainable, a sufficiently high rate of submission and/or a low cost of data integration are required. This model works well for repositories where the main goal is to preserve data rather than add value through curation and integration with related datasets, as the built-in financial incentive encourages a repository to maximize the number of submissions independent of degree of data curation, data type or knowledge domain. It works less well for curated community databases that specialize in integration of content for specific knowledge domains because the volume of data is lower and the cost of integration is higher.

An alternative to the fee for submission model is the fee for access model. In this model, user fees may take the form of memberships or subscriptions. A well-established example is the Inter-university Consortium for Political and Social Research (ICPSR), which charges a membership fee for access to a data repository for the social sciences (https://www.icpsr.umich.edu/icpsrweb/landing.jsp). Member fees support the activities of the group and members have privileged access to repository data and tools. Another example is the Kyoto Encyclopedia of Genes and Genomes (KEGG) (10), which allows free browsing of web pages but requires a subscription for academic users to access the ftp site. Commercial users must pay for both browsing and downloading KEGG data. KEGG began requiring academic subscriptions for access to ftp download when their funding was cut as a means to keep the project open and updated. In 2014, KEGG received new grant support covering about a third of their operational costs (http://www.genome.jp/kegg/docs/plea.html) and they continue to rely on academic and commercial subscriptions to cover the remainder of their costs. Another quasi-open access variation is the ‘freemium’ model, whereby access to ‘basic’ data or features is free and users pay extra for additional features such as proprietary data analysis tools (e.g. Genevestigator; www.genevestigator.com) or other premium features (e.g. FigShare; www.figshare.com). A third user-based funding model, voluntary contributions, is how many public radio or television stations are funded. This model has been adopted by arXiv, a well-known repository for physics and mathematics article preprints (http://arxiv.org/). However, in this case, even with a large donor base, contributions alone are not adequate. Membership dues cover about a third of arXiv’s, operating costs,; the rest comes from its host institution (http://arxiv.org/help/support).

Here, we present our experience with finding user-based support for The Arabidopsis Information Resource (TAIR), which demonstrates that subscription fees can be a viable alternative source of funding to sustain databases. TAIR was established in 1999 as a model organism database for *Arabidopsis thaliana* (11). TAIR is used by over 60 000 researchers worldwide each month and is the main source of manually curated data for Arabidopsis (12). TAIR curators extract and integrate a range of experimental data from the primary literature including gene names, gene function, gene expression patterns, alleles and phenotypes (13–15). Curators continuously update information about Arabidopsis genes using a combination of computational text-mining methods and manual curation of the primary literature. They assign experimentally based functions to genes using Gene Ontology (GO) annotations, process and perform quality control on data submissions, resolve issues of gene nomenclature and help design the systems and tools to expose and analyze the data in TAIR (16). TAIR curators prepare customized data sets for users on request and also serve the research community by providing essential helpdesk functions, answering dozens of questions each month about how to access, use and interpret Arabidopsis research data. Curators also present workshops at international plant biology research conferences to introduce researchers to the available data and tools. In a partnership with the Arabidopsis Biological Resource Center, TAIR provides integrated stock search, browse and order functions to the community (17). From its inception until 2013, TAIR was funded almost exclusively by three consecutive grants from the National Science Foundation’s Division of Biological Infrastructure (NSF-DBI). In 2009, TAIR staff learned that, although TAIR had been granted a renewal, its NSF funding would be cut by 25% each year, terminating in 2013 (18). Faced with the prospect of losing funding for its reference database, the community rallied and proposed a new kind of infrastructure project capable of distributing the cost and work of collecting and maintaining datasets and tools over many groups and countries (19). This new project, funded by the NSF and the Biotechnology and Biological Sciences Research Council, was officially launched in 2014 as Araport [https://www.araport.org/; (20)]. Araport was designed as framework that enables the research community to develop and contribute modules either in the form of data sets, applications that operate on the data, or both (20). It relies on a data federation and community model in which independent projects are responsible for obtaining funding and generating the data that are then aggregated and displayed within the portal. Araport’s data federation approach provides a very useful new collection of data types and tools but does not replace TAIR’s essential function—the addition of new knowledge extracted from research articles to the gold standard gene function data set for Arabidopsis (12). Indeed, TAIR is the main source for curated gene summaries, gene names, Plant Ontology (PO) annotations and phenotypes in Araport. The impact of this gold standard data set extends well beyond the Arabidopsis community, as Arabidopsis research is the source of many fundamental discoveries in plant biology and continues to be a widely used reference for understanding plant gene function (21, 22). TAIR provides an easily accessible, centralized location where researchers can go to mine these essential experimental results and apply them towards other organisms.

The plant biology community’s continuing need for TAIR’s services and the lack of any grant-based funding options eventually convinced the four remaining staff members to pursue alternative funding strategies to support TAIR. After careful consideration of several different funding strategies, TAIR began collecting subscription fees in January 2014, and is now supported almost exclusively by subscriptions. Transitioning to a new funding source required a new infrastructure, relevant new expertise and new software. With funding from the Sloan Foundation, we are now poised to extend our user funding model to other databases as a means to entirely support or supplement funding for ongoing activities such as biocuration.

## Materials and Methods

### Founding phoenix bioinformatics to support sustainable databases

Having made the decision to continue the TAIR project, four staff members (Dr Huala, Dr Berardini, Dr Li and Dr Muller) founded Phoenix Bioinformatics, a 501(c)(3) nonprofit, with the primary goal of finding alternative, sustainable funding mechanisms for databases and other infrastructure projects serving the academic research community (www.phoenixbioinformatics.org). We realized that TAIR was not the only resource facing this problem and that there was a need for an organization specifically dedicated to database sustainability that could serve as an umbrella entity, providing infrastructure and support for orphaned projects. Phoenix launched in September 2013 with TAIR as its first project. To help pursue its mission, Phoenix recruited a diverse and highly skilled board of directors with expertise in publishing, informatics, finance and nonprofit management.

### Developing a subscription fee model for TAIR

After carefully researching and modeling different user fee alternatives, Phoenix concluded that the collection of subscription fees was the most likely path towards achieving a level of sustainable funding that would allow TAIR to continue to provide the high-quality data and analysis that researchers require. Our previous experiment with voluntary sponsorship support from companies suggested that funding from voluntary participation always lags behind a mandatory requirement and is also inherently less fair, rewarding free riders and penalizing good citizens. Requiring researchers to pay for data submission (as in the PLoS or Dryad model) did not appear likely to generate enough revenue to support TAIR’s curation efforts, as TAIR curators enter the majority of data, and only a small fraction is submitted by the community. In the absence of a data submission requirement enforced by funding agencies, journals or host institutions, asking researchers to pay to submit data would likely further reduce the number of submissions and fail to bring in sufficient funding to support the project. Also, in the absence of a universal data submission requirement, a pay to submit model is also likely to be somewhat random in the type and quality of data collected, resulting in an arbitrary sampling of available data rather than a curated collection of complete and integrated datasets with the widest possible utility to the research community.

In devising a subscription-based funding strategy for TAIR, we adhered to a set of guiding principles aimed at maximizing wide data availability, sharing and reuse, while providing sufficient revenue to maintain the level of quality that TAIR’s users expect. These principles are: (i) to facilitate reuse by other repositories, TAIR data would be made free to all after 1 year; (ii) subscriptions to access more recent data should be affordable and offered at a variety of levels to maximize options and coverage of researchers; (iii) to support occasional users, researchers should be able to access a few pages per month of recent data without subscribing; (iv) TAIR should be free for students enrolled courses that use TAIR as part of the curriculum; (v) access should be free to the lowest income countries.

To achieve our goal of facilitating broad access, we decided to offer a range of subscription options. Therefore, academic and nonprofit researchers can subscribe at the country, consortium, institution or individual level. The broadest coverage comes from government subscriptions, which provide access to all academic researchers within the country and are paid for by government agencies. Institutional or consortium subscriptions, which, like journal subscriptions, are typically paid for by libraries, provide access to all faculty, staff and students at the covered institution(s). Researchers at nonsubscribing institutions have the option to subscribe as individual researchers, with a discounted rate for two or more subscriptions purchased together. Depending on the funding source, researchers may be able to recover the cost of their TAIR subscription from their grants.

In order to establish a fair and reasonable pricing strategy for academic institutions, we researched different subscriber models and pricing, mostly from the publishing industry. We found that most academic journals charged subscriptions based upon the Carnegie classification (http://carnegieclassifications.iu.edu/), in which the largest (based on full time equivalent attendance, [FTE]) and more research intensive institutions pay a higher rate than small, primarily teaching colleges do. However, the Carnegie classifications do not apply to non-US institutions, making them a less than ideal tool for a database with a majority of international users. Additionally, nearly all the US institutions using TAIR fall into the research university category of Carnegie classification (very high or high activity; data not shown). Therefore, we investigated pricing based on usage as an alternative and potentially fairer way to allocate the cost of supporting TAIR over a global set of academic institutions with widely varying usage patterns.

**Figure 2.**
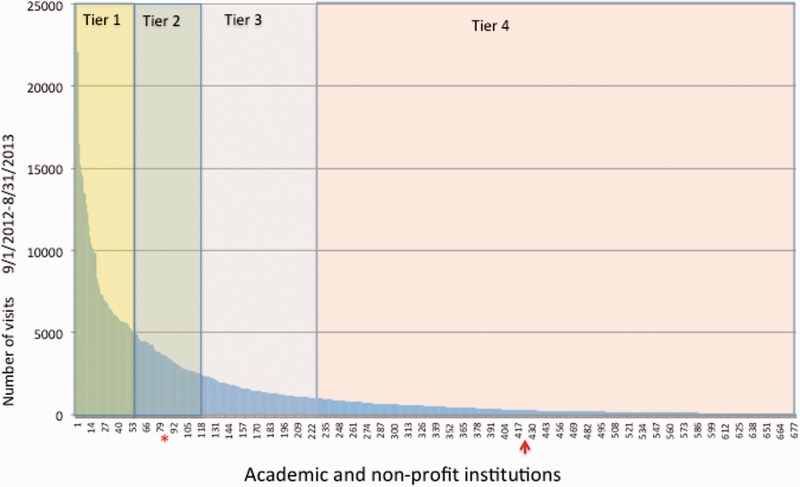
Usage based classification of academic/nonprofit institutions. A histogram showing the distribution of academic and nonprofit institutions grouped into tiers from highest (Tier 1) to lowest (Tier 4) usage. Usage is based on the number of visits tracked by Google Analytics over the period of 1 year, from 1 September 2012 to 31 August 2013. Data for institutions with more than one network domain are combined into a single data point. From a total of 685 institutions, 57 are Tier 1, 63 are Tier 2, 109 are Tier 3 and 456 are Tier 4. Florida State University (arrow), a large 4 year university has low usage (Tier 4) whereas Dartmouth College (asterisk), a medium size 4-year college, has relatively high usage. Both are research intensive institutions.

We used Google Analytics to examine TAIR usage patterns over a period of several years. We gathered information about the frequency of access, geographic distribution of users, most highly viewed pages and most common browsing paths taken within the site. We found that usage (defined as number of sessions) did not necessarily correlate with institutional size based on FTE. Some large universities had relatively low usage (e.g. Florida State University, large 4 year; Figure 2) and some smaller universities, with large plant biology departments, had relatively very high usage (e.g. Dartmouth, medium 4 year; Figure 2). We then divided the academic institutional usage into four tiers from highest (Tier 1) to lowest usage (Tier 4) and assigned subscription rates based upon these tiers. For corporate subscribers, rates were based on the size of the company calculated by revenue, similar to standard publisher models.

To establish the price for each academic tier and for other types of academic subscriptions for individuals, consortia and countries, we estimated the fraction of the total TAIR usage represented by each entity and used that fraction, applied to the total revenue required to sustain TAIR, as the basis for the subscription price. We then adjusted the prices to provide a discount for larger subscribing entities. We did this for two reasons: (i) the effort needed for us to obtain and manage a subscription for a large entity like a consortium or country is less than the effort required if the academic institutions or all individuals within them subscribed separately; and (ii) we wanted to encourage country and institutional subscriptions because they would cover individuals who might not subscribe on their own, such as rotating graduate students, undergraduates and researchers in labs or institutions with more limited budgets. In this way, we were able to use the price structure to further our aim of maintaining broad access to TAIR.

To enable subscription collection, we established systems for account management, licensing and payments and defined standard protocols for tracking and reporting usage. We built a robust, customized account management platform using a Salesforce, an industry standard customer relationship management system available free of charge to nonprofit organizations, allowing us to track contacts with potential subscribers including researchers, companies and university librarians. We employed different strategies to inform our community about the upcoming access changes including email, social media, posting on websites and conferences. We opted to do a slow rollout of the subscriptions, first to commercial users and then to academic and nonprofit researchers to give our users ample time to prepare for the change and make any necessary budget adjustments and requests to libraries. Finally, we enlisted appropriate legal expertise to draft academic and commercial licenses and updated terms of use for the database.

### Subscription management system software technology

The subscription plan we chose to adopt includes unlimited access to TAIR’s tools and recently curated data for subscribers. Nonsubscribers retain limited access to tools and recent data and unlimited access to older data, in accordance with our guiding principles. Therefore, we needed a way to grant subscribers full access while still offering limited access to nonsubscribers. This required implementing software capable of providing TAIR access based on subscription status. TAIR required a complex and intelligent subscription software system capable of controlling access based upon login and IP address, providing metered access for TAIR pages to enable free access for occasional users, and providing open access to DNA and seed stock search, details and ordering functions for our partner, the Arabidopsis Biological Resource Center. We investigated the possibility of adopting commercial paywall services used by news and magazine publishers (e.g. Tinypass, Press Plus or Piano Media), but given our specific requirements, the initial and ongoing costs of the commercial services and our desire to find a general solution that could also serve other repositories, we opted to develop our own custom subscription management system. Phoenix’s initial implementation of the TAIR subscription management proxy server is a Java Enterprise Edition web application running virtual machines at The Texas Advanced Computing Center. The subscription management software stack uses Apache and Tomcat middleware and a back-end Oracle database to maintain subscriber data, metering tracking, content specifications and usage statistics. The system also provides a REST API to support user interface applications written in JSF and JQuery as well as internal operations for the proxied system (TAIR).

**Figure 3.**
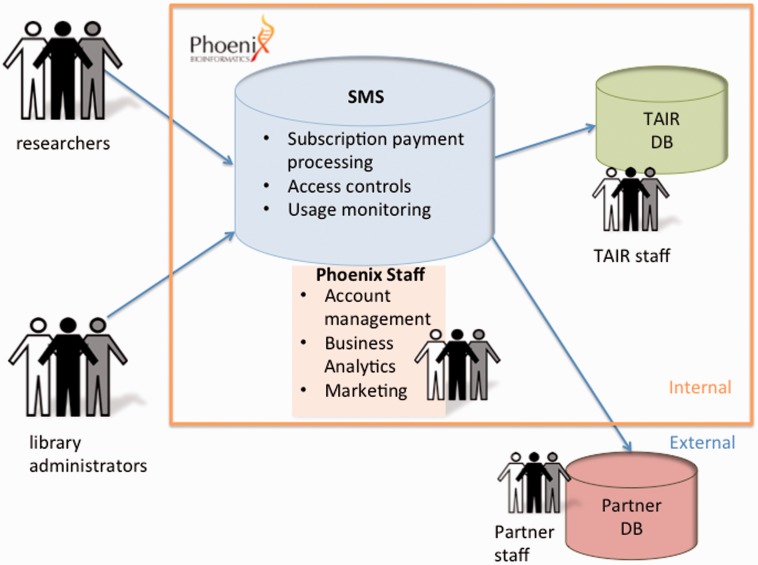
Subscription management services. The subscription management service software layer serves as an interface between users (researchers and librarians) and databases hosted by Phoenix (e.g. TAIR) or external databases hosted by partners. The software functions include subscription enrollment and payment processing, access control and usage monitoring. It can be customized to accommodate variable metering limits, different user fee models, and display of partner logos. Phoenix staff functions include account management, marketing and business analytics for databases hosted and managed by Phoenix (e.g. TAIR) as well as partner databases that continue to be hosted and managed externally, operating as independent entities with their own staff and infrastructure.

In March 2015, Phoenix received a grant from the Sloan Foundation to enhance the technology behind TAIR’s subscription funding model and to apply that model (and potentially other user fee models) to additional database partners beyond TAIR. The award from Sloan’s Digital Information Technology program supported the development of a next-generation, flexible and customizable technology platform capable of serving other databases and research resources wishing to shift to user-based funding. The new platform is an API consisting of modular, scalable services built on a cloud infrastructure housed at Amazon Web Services, providing a set of secure REST web services using HTTP and JSON formats over SSL connections. Services available through the API include (i) authentication, authorization and attribution; (ii) subscription and payment support; (iii) account management and administration tools; and (iv) tools for the integration of new partners (Figure 3). The design permits customization of user interfaces including branding and partner-specific marketing language. It also includes subscriber account management functionalities such as renewal requests, updating librarian or consortium contact information, adding/removing subscriptions and adding/removing IP addresses. Additional features including usage reporting are currently under development.

## Results and Discussion

### TAIR subscription revenue is sustainable and international

TAIR has successfully made the transition to a subscription-based funding model that provides long-term continuous support and distributes the cost of maintaining TAIR over many countries. Within 1 year after beginning to collect subscriptions, TAIR’s annualized subscription revenue reached $860000, an amount approaching the annual budget when the project started in 1999. With that funding we have been able to maintain our rate of literature curation, upgrade our software systems and make other long-delayed improvements and bug fixes.

**Figure 4.**
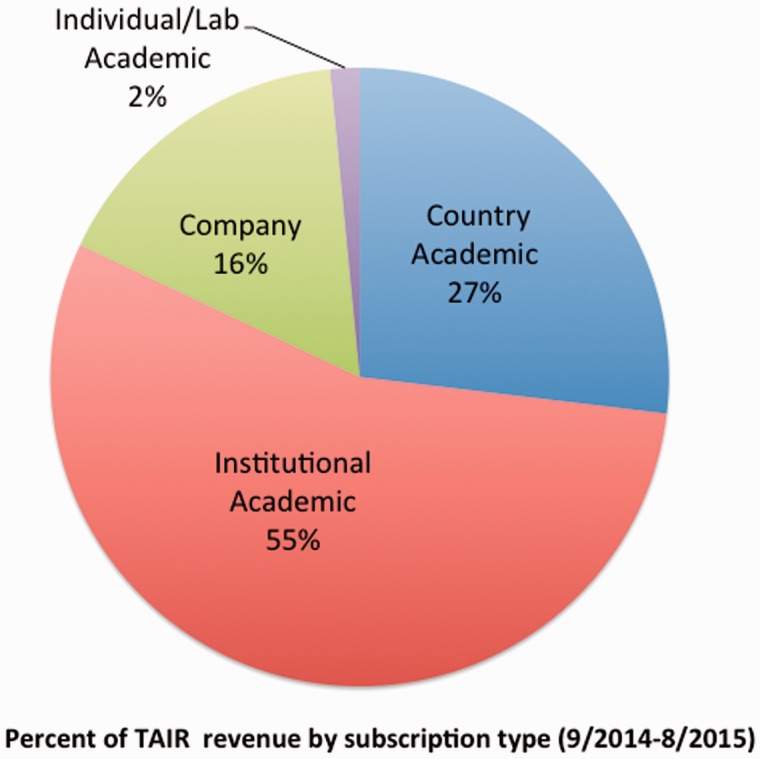
TAIR subscription distribution by type. The majority of revenue (55%) comes from institutional academic subscriptions followed by country/government academic subscriptions (27%), and companies (16%). Individual subscribers contribute about 2% of revenue.

As of October 2015, TAIR subscribers include 2 countries (China and Switzerland), 4 academic consortia, 146 academic/nonprofit institutions and 240 individuals. Corporate subscribers include five major agricultural biotechnology companies and five smaller companies. Additionally, we have provided over a dozen free institutional subscriptions for teaching purposes. As of August 2015, the bulk of TAIR subscription revenue comes from the institutional (55%) and government (27%) academic subscriptions (Figure 4), followed by corporate subscriptions (16%). Individual academic subscriptions have doubled since March 2015, and as of October 2015, they account for 2% of revenue. Institutional subscriptions are distributed across the four tiers (Tier1 = 26, Tier 2 = 35, Tier 3 = 39 and Tier 4 = 46). Consortium subscribers include the University of California system and the Max Planck Society Institutes. To date, the renewal rate for academic institutions has been 98%. We estimate that just over half of our users are now subscribed. For the month of September 2015, excluding search bots, 6316 of the 12 691 IP addresses that accessed TAIR were registered institutional subscriber addresses. That number does not include accesses by researchers with personal, login-based subscriptions. Although we have been quite successful in acquiring and retaining subscribers, these statistics show that there are still more who could subscribe but as yet do not. We are continuing to reach out to researchers and librarians at nonsubscribing institutions to ensure as wide access to TAIR as possible.

**Figure 5.**
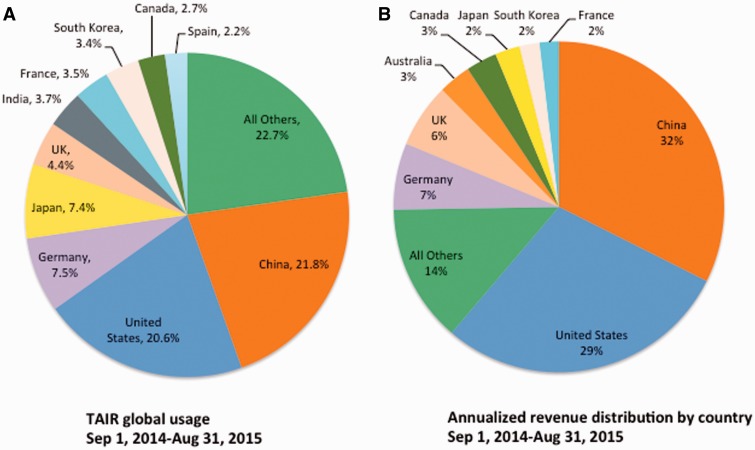
Global Distribution of TAIR users and revenue. **(A)** Global distribution of TAIR users for the period of 1 year from (1 September 2014 to 31 August 2015), countries with <2% of usage are pooled (All Others, 22.7%). **(B)** Global distribution of revenue. Annualized revenue from institutional/government subscriptions for the same time period. Note that this does not reflect the total distribution by country because individual subscribers are not included in the figure.

As expected, subscriptions have had the effect of distributing the cost of maintaining TAIR across the world. Whereas our previous grant funding was entirely US based, the distribution of revenue by country now more closely reflects the composition of TAIR’s global user community (Figure 5A). Although we did not expect the distributions to have a one-to-one correspondence, the profiles are similar. For example, the two countries with the greatest usage, China and the USA (21.8 and 21%, respectively) contribute the majority of subscription funding (a combined 61%) (Figure 5B). Some countries’ contributions are lower than their usage. Reasons for this discrepancy are both logistical and financial. For example, in some countries, libraries prefer to purchase subscriptions through subscription agents and our initial strategy of approaching librarians directly in such countries did not have wide success. Unfavorable currency exchange rates, budget constraints or library policies may also negatively influence institutional subscription rates in specific countries. In such cases, individual subscriptions provide an alternative access route for researchers. In Japan, one of the ten countries with highest TAIR usage, researchers are primarily subscribing at the individual level.

We are continually evaluating and refining TAIR’s subscription model as we gather feedback from our stakeholders. For example, in our interactions with librarians, we have heard concerns about the usage tier model and the inherent uncertainty of pricing from year to year if usage patterns change. Therefore, we began offering multi-year subscriptions, allowing institutions to lock in a price and removing fiscal uncertainty over a set time period. Thirty-two of our institutional licenses are now multi-year agreements.

### Impacts of the subscription model on data reuse, access and submission

A frequent criticism of subscription funding is the potential to negatively impact data reuse (3, 5, 8). TAIR’s data release policy is designed to minimize this drawback by carefully balancing subscriber-only privileges that serve as incentives to subscribe with unrestricted access to foster wide dissemination and data reuse. Curated data are updated weekly and are available to subscribers. After the data have been in TAIR 1 year, they are released on a quarterly basis and are freely available for anyone to download (https://www.arabidopsis.org/download/index-auto.jsp?dir=/download_files/Public_Data_Releases). The exception is the full set of TAIR GO annotations, which is released monthly to the GO consortium site (http://geneontology.org/page/download-annotations), where it can be downloaded and reused by other data resources such as NCBI (http://www.ncbi.nlm.nih.gov/genome/4) and the Universal Protein Resource (UniProt; www.uniprot.org). Since the first quarterly data release in December 2014, TAIR’s public release pages have been accessed nearly 1000 times. Reuse of TAIR data appears to be robust, with external resources continuing to update their databases with TAIR’s freely released, year-old data including: Araport (gene names, descriptions, alleles and phenotypes), Salk SIGNAL T-DNA insertion database (http://signal.salk.edu/cgi-bin/tdnaexpress; gene names and descriptions), the PO database (http://www.plantontology.org/; gene names, descriptions, and PO annotations for gene expression), and the Bio-Analytic Resource for Arabidopsis (http://bar.utoronto.ca/; GO annotations, gene names, gene descriptions). Subscription models can potentially have negative effects on data interoperability if the flow of data is halted to protect commercial interests (3) . However, TAIR is a non-profit and our motivation is to continue to provide valuable data and services to the community at a reasonable cost, not to make a profit. The imposed year-long delay in the release of some of the value-added data at TAIR, allows us to support continuous data generation while still maintaining interoperability with public databases.

**Figure 6.**
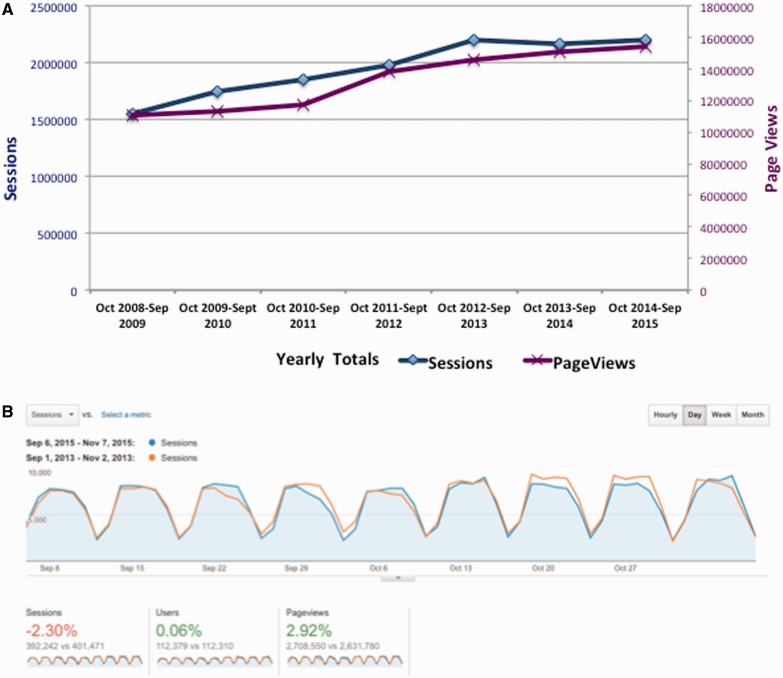
TAIR historical and recent usage trends. **(A)**. Annual usage of TAIR database shown as both number of sessions (blue, left axis), and total number page views (purple, right axis). **(B)** Close up snapshot comparing pre- and post-subscription usage. Google Analytics dashboard showing the number of sessions and page views over an 8-week period from September to November 2015 and an equivalent period in 2013. Dates were adjusted to include the same number of weekday and weekend days for each period. Similar to the year-by-year comparison in panel A, the number of sessions (dark blue) shows a slight decrease while the number of page views shows an increase (dark orange).

Another concern is that a subscription model would decrease use of the database. To date, we have seen relatively minor changes in TAIR usage since moving to subscription funding. Based on data from Google Analytics, the number of page views has increased each year. Sessions increased initially but leveled off in the past 2 years (Figure 6A). Figure 6B shows a snapshot of pre- and post-subscription time intervals, comparing user data over a similar 8-week time period in 2013 (before subscriptions were required) and 2015 (after subscriptions). The trend is similar to the general pattern seen in panel A (September 2012 to October 2013 versus September 2014 to October 2015) with a slight decrease in the number of sessions and an increase in page views. In 2013, there were 401 471 sessions from September 1 to November 7, while in 2015 there were 392 242 sessions. A slight decrease is usage is not unexpected as not all users are covered by subscriptions at this point in time, though we continue to improve our coverage each month. Interestingly, while users are initiating fewer sessions they are accessing more pages (data) per session. We will continue to carefully monitor usage to assess the impacts of subscriptions on data access.

We were also curious to see how the change might impact the frequency of data submissions (such as gene function annotations) via our community curation pipeline (23) in which TAIR accepts functional annotation and gene expression annotation data from the community. Researchers can use the TAIR Online Annotation Submission Tool to curate papers and annotate gene names, descriptions, gene function using GO terms and gene expression using PO terms (23). Before they are incorporated into TAIR, the annotations are reviewed and sometimes modified or supplemented with additional annotations by TAIR curators. So far, we have not seen any negative effect on data submissions. In the year prior to implementing the academic subscription requirement, TAIR processed annotations from 97 community members (dates 1 April 2013 to 31 March 2014). In the following year (1 April 2014 to 31 March 2015) we processed annotations from 123 community members.

### Extending the paradigm to other databases, challenges and opportunities

Phoenix’s success in transitioning TAIR to user based funding is encouraging and suggests that user funding could be applied to other databases as well. However, any database contemplating a move to subscription-based funding needs to consider many factors before making that transition. Some of the factors that have likely contributed to TAIR’s success with subscriptions are: (i) it is a well-established resource with a large, globally distributed user base, and (ii) it has a unique niche within the Arabidopsis data ecosystem as the primary resource providing manual curation and data integration. Collecting subscription fees may be significantly more challenging for databases with smaller user communities or those without unique attributes that differentiate them from other resources. If the community is small, it might be hard to generate sufficient revenue to maintain a database. For newly established databases, requiring subscriptions might slow the growth of their user community and, again, result in too little revenue for sustainability or further growth. For some databases, other kinds of user funding such as voluntary contributions, a charge for data submission or a charge to download data might be a more appropriate choice. These funding mechanisms may be able to provide bridge funding between grants (24, 25) or provide partial funding, supporting activities that have typically been hard to fund with competitive grants, such as biocuration and database maintenance. For other databases that have lost grant funding, the best option may be to archive the data in an institutional repository or have it absorbed into a resource with more long-term viability, rather than seeking user-based funding.

Perhaps the most significant barrier to adoption of a subscriber model is the concern that some researchers will lose access to important data or tools (5, 9, 26). By carefully balancing revenue versus access for all segments of the user community, this drawback can be minimized. To maintain broad access, it is essential that the cost for users is affordable and that alternative access points, such as metered access for occasional users or free access for teaching and low-income countries, are provided.

Another barrier may be the cost of making the changes required to move to a user-based funding model. If the effort required to make such a transition can be reduced, it may become feasible for more databases. The researchers using such databases would benefit by continuing to have the database available and up to date, and subscription costs could also be reduced if the change can be made with a minimum of effort and expense. As part of Phoenix’s mission to make databases sustainable and data broadly accessible, we want to make it easier for other research infrastructure projects to move to sustainable funding models. For that reason, in addition to the subscription management software, Phoenix also offers help with business planning, user analytics, licensing, marketing and sales, areas in which most academic databases have little expertise (Figure 3).

### User funding is a viable component of a broad toolkit for ensuring database sustainability

Secure funding plays an essential role in assuring sustainability of data repositories but is not the only necessary ingredient. Reducing costs and increasing efficiency can help databases make better use of limited funding, whatever its source. Significant efforts in this area include (i) increasing the speed and accuracy of computationally assisted curation through the efforts of initiatives such as BioCreative (http://www.biocreative.org), (ii) reducing database and tool development costs through the creation of shared, distributed software, infrastructure and tools (4, 5, 26) and (iii) more public education and outreach about the importance of curation and the costs of maintaining and improving essential community resources (15, 27). Recently two significant initiatives have been launched to address these broad challenges. The US National Institutes of Health Big Data to Knowledge [BD2K; (28) and European ELIXIR (29)] projects are focused on establishing sustainable data ecosystems in part by creating shared infrastructure, resources, methods and tools to better access analyse and understand big data. These initiatives have great potential to catalyse changes in the way information is stored, accessed and shared, and to contribute to overall sustainability of the big data ecosystem. It remains to be seen how these advances will impact community databases. BD2K is piloting the use of cloud hosting for community resources within the NIH Commons to assess potential for greater efficiency and lower cost. If such an approach is workable, it offers some hope for greater sustainability, at least for biomedical databases that fit within the NIH mission.

The research community is a relatively untapped resource that has great potential to increase the efficiency and sustainability of databases. To reduce the cost of curation, more effort must be devoted to recruiting, training and motivating researchers to curate and submit their own data (5). Several model organism databases, including TAIR and PomBase, have established pipelines and software to facilitate literature curation by the community (23, 30). In Flybase, users can perform the initial triage by associating data objects such as genes to their articles (31). These tools can reduce curation costs for a database, but as yet, none have replaced the need for dedicated curators. Authors are more likely to contribute when a curator actively solicits their annotations versus simply having submission software available. Also, community submissions generally require some review and revision by curators. Journals can also help reduce curation costs by requiring authors to associate unique identifiers of genes, mutant lines or other biological materials to their articles prior to article acceptance. Currently, tagging of data objects in articles is largely voluntary (32–34). There is also a need to establish curation and data format standards, similar to those for sequences and microarrays, for a wider range of data types so that data can be more easily discovered and interconnected (26). Databases and funders will need to identify the proper incentives to encourage researchers to annotate and curate their work, such as documenting increased citation rates for curated and annotated articles (35) or providing DOIs for annotations submitted by a researcher that can be added to that researcher’s curriculum vitae as a type of publication. Taken together, these efforts can result in data resources that are stable, up to date, and effective in enabling scientific progress.
